# Daphnetin ameliorates acute lung injury in mice with severe acute pancreatitis by inhibiting the JAK2–STAT3 pathway

**DOI:** 10.1038/s41598-021-91008-6

**Published:** 2021-06-01

**Authors:** Shujun Yang, Yaodong Song, Qiaofang Wang, Yanna Liu, Zhongwei Wu, Xiaojia Duan, Yan Zhang, Xiuqian Bi, Yuanzhang Geng, Sanyang Chen, Changju Zhu

**Affiliations:** 1 Department of Emergency, The First Affiliated Hospital of Zhengzhou University, No 1 Eastern Jianshe Road, Zhengzhou, 450052 Henan China; 2 Henan Medical Key Laboratory of Emergency and Trauma Research, Henan, China; 3 Key Laboratory of Hepatobiliary and Pancreatic Surgery and Digestive Organ Transplantation of Henan Province, Zhengzhou, Henan, China

**Keywords:** Biochemistry, Chemical biology, Drug discovery

## Abstract

Severe acute pancreatitis (SAP) is often associated with pulmonary inflammation leading to acute lung injury. Daphnetin, a natural coumarin derivative, has been reported to exert anti-inflammatory effects. Here, we explored the effect and possible mechanism of daphnetin in a mouse model of SAP-associated lung injury induced by an intraperitoneal injection of l-arginine. The severity of pancreatic and lung injury is determined by histology and its score. Immunostaining of inflammatory and apoptotic cells was used to demonstrate lung tissue inflammation and apoptosis; ELISA analysis of serum and tissue cytokine levels; and western blotting and immunohistochemical staining for the activated Janus kinase 2 (JAK2)–signal transducer and activator of transcription protein 3 (STAT3) signalling pathway in lung tissues. Daphnetin pretreatment significantly reduced SAP-induced pancreatic and lung tissue damage, reduced interleukin-6 and tumour necrosis factor-α concentrations in both serum and lung tissues, reduced serum amylase and myeloperoxidase activities, and reduced macrophage (CD11b) and neutrophil (Ly6G) infiltration and cell apoptosis in the lung tissue. Moreover, SAP-induced phosphorylation of JAK2 and STAT3 in the lung tissue was also significantly diminished by the daphnetin pretreatment. These results indicated that daphnetin reduces SAP-associated lung tissue damage, likely by inhibiting the activation of JAK2–STAT3 signalling.

## Introduction

Severe acute pancreatitis (SAP) is an inflammatory disease characterized by a complicated aetiology, rapid progression, and poor prognosis^1,2^. SAP may lead to multiple organ dysfunction, of which SAP-associated acute lung injury (PALI) is the most common complication^3^. PALI is associated with high morbidity and mortality rates and is responsible for 60% of deaths in the first week after a SAP diagnosis^4^.

Inflammation plays a well-characterized role in the occurrence and development of PALI. SAP induces the production of pro-inflammatory mediators, including chemokines and cytokines such as interleukin-6 (IL-6) and tumour necrosis factor-α (TNF-α), and increases the tissue infiltration and activation of macrophages (CD11b) and neutrophils (Ly6G), leading to pancreatic tissue necrosis^5,6^. IL-6 regulates the infiltration of neutrophils and promotes inflammation by binding to the IL-6 receptor and activating a signalling pathway involving Janus kinase (JAK) and signal transducer and activator of transcription protein 3 (STAT3)^5,6^. In the lung, SAP-associated release of pro-inflammatory mediators, excessive endothelial cell damage, and activation of multiple inflammatory pathways lead to increased pulmonary microvascular permeability, resulting in increased penetration of neutrophils into the lungs and perpetuation of the inflammatory cascade^8,9^. Moreover, the release of numerous inflammatory factors induces the apoptosis of pulmonary microvascular endothelial cells, which results in endothelial barrier dysfunction^10^. Despite considerable investments in time and effort into understanding the development of PALI, effective treatments are still lacking^10^. Thus, studies exploring the molecular mechanisms underlying PALI are a great need, thereby enabling the development of novel potential treatments.

The JAK–STAT signalling pathway plays an important role in regulating diverse cellular processes, such as apoptosis, inflammation, and the oxidative stress response^11^. The pathway is activated by a range of cytokines, growth factors, and proinflammatory mediators^11^. Engagement of the corresponding receptors induces the phosphorylation and activation of JAK, which then phosphorylates STAT proteins, promoting their translocation to the nucleus and the regulation of target gene transcription^12,13^. Evidence from many clinical and preclinical studies has suggested that the JAK2–STAT3 pathway plays a pivotal role in lung injury^14^.

Daphnetin (7,8-dihydroxycoumarin), a natural coumarin derivative, is present in extracts of *Daphne odora* var. marginata^15^. Daphnetin possesses numerous pharmacological properties, including analgesic, anti-inflammatory, and anti-oxidant effects, and has been widely used in the clinic to treat diseases such as rheumatoid arthritis, thromboangiitis obliterans, and coronary heart disease^16–18^. Daphnetin has also been suggested to protect rats from acute pancreatitis induced by sodium taurocholate^19^ and to exert an anti-inflammatory effect on an endotoxin-induced model of lung injury^20^. It also alleviates acute liver failure by inhibiting NLRP3, MAPK and NF-κB^20^. Taken together, these observations suggest that daphnetin may be useful for the treatment of PALI.

Here, our study showed that a daphnetin pretreatment reduced SAP-induced pancreatic and lung tissue damage. And we found that the reduction in lung injury might be due to the inhibitory effect of daphnetin on the activation of the JAK2-STAT3 signalling pathway in lung tissue. These findings may partially describe inflammatory responses involved in PALI, which improves our understanding of the pathogenesis and offers potential benefits for disease treatment.

## Materials and methods

### Reagents

Daphnetin was purchased from Selleck (S2554). The daphnetin preparation used in the present study was 99.23% pure. l-arginine and BCA protein assay kit was purchased from Solarbio Science and Technology (A0013-25g, PC0020). RIPA lysis buffer, phosphatase inhibitor cocktail, and horseradish peroxidase (HRP)-conjugated secondary antibodies were purchased from Beyotime Biotechnology (P0013B, P1081, A0208). Primary antibodies against glyceraldehyde 3-phosphate dehydrogenase (GAPDH), JAK2, and STAT3 were purchased from Proteintech (10494-1-AP, 17670-1-AP, 10253–2-AP), and primary antibodies against the phosphorylated forms of JAK2 and STAT3 (p-JAK2 and p-STAT3) were obtained from Cell Signaling Technology (4406T, 9145T). Serum amylase and lipase enzyme-linked immunosorbent assay (ELISA) kits were purchased from Shanghai Enzyme-linked Biotechnology (ml037819, ml064283). Myeloperoxidase (MPO) ELISA kits were purchased from Cloud Clone (SEA601Mu), and TNF-α and IL-6 ELISA kits were purchased from Proteintech (KE10002, KE10007). Primary antibodies against Ly6G and CD11b, Cy3-conjugated goat anti-rabbit IgG secondary antibody, and terminal deoxynucleotidyl transferase dUTP nick end labeling (TUNEL) apoptosis detection kit were purchased from Servicebio (GB11229,GB11058,GB21303, G1501).

### Animals

Twenty-four male C57BL/6 mice (aged 6–8 weeks and weighing 16–20 g) were purchased from the Animal Experimental Center of Zhengzhou University. All animals were housed under standardized conditions in a controlled environment of 22 °C on a 12 h light–dark cycle and were allowed free access to food and purified water. Experiments were approved by the Ethics Committee of Scientific Research of the First Affiliated Hospital of Zhengzhou University (review number 2019-KY-140) and were conducted in accordance with institutional instructions and regulations.

### Animal model of PALI

Mice were fasted (free access to water) for 12 h and then assigned to four groups (n = 6): (1) a control (CON) group (l-arginine vehicle + daphnetin vehicle), (2) SAP group (l-arginine + daphnetin vehicle), (3) DAP group (l-arginine vehicle + daphnetin) and (4) SAP + DAP group (l-arginine + daphnetin).

The SAP model was established by administering two intraperitoneal injections (1 h apart) of l-arginine at a dose of 4 g/kg body weight (diluted in saline, pH 7.0) or the same volume of normal saline (vehicle). Daphnetin was dissolved in 5% dimethyl sulfoxide (DMSO) according to the manufacturer’s instructions, and a dose of 4 mg/kg body weight was administered by intraperitoneal injection 30 min before the injection of l-arginine or vehicle. The SAP group was injected with an equivalent volume of 5% DMSO before the l-arginine injection. After 24 h, the mice were anaesthetized with an intraperitoneal injection of 1% pentobarbital sodium, and blood samples were collected using the orbital venous plexus bleeding method. The blood samples were left undisturbed for 10 min and then centrifuged at 3000 rpm for 10 min at 4 °C. The serum was collected and stored at − 80 °C until analysis. Mice were then sacrificed, and their lungs were removed and divided into two portions: one was stored at − 80 °C and the other was fixed with 4% formaldehyde for subsequent embedding in paraffin and sectioning. The pancreas was removed and fixed with 4% formaldehyde for the histological examination.

### Histology

Pancreas and lung tissues were fixed with a 4% paraformaldehyde solution for 2 to 3 days and embedded in paraffin. The blocks were cut into 4-μm sections, stained with haematoxylin and eosin (HE), and evaluated using an optical microscope. Ten random fields (400 × magnification) were selected and evaluated by two experienced pathologists who were blinded to the experimental treatments. The severity of acinar cell injury was evaluated on a semi-quantitative scale based on oedema, inflammatory cell infiltration, and acinar necrosis, as described by Schmidt et al. (1992)^22^. The severity of lung tissue injury was based on the degree of pulmonary cell edema, hemorrhage, and inflammatory cell infiltration refering to MO, O. et al.^23^.

### ELISA

Serum amylase, serum lipase, serum and lung IL-6, serum and lung TNF-α, and lung MPO levels were determined using specific ELISAs according to the manufacturers’ instructions.

### Immunofluorescence staining

The expression of CD11b and Ly6G, which indicate the presence of macrophages and neutrophils respectively, were analysed by immunostaining lung sections. Sections were dewaxed, hydrated, and treated with EDTA-containing antigen retrieval buffer (pH 8.0) in a microwave oven. The tissue was encircled using an immunohistochemical pen and blocked with 5% bovine serum albumin at room temperature. The slides were incubated overnight at 4 °C with primary antibodies against CD11b (1:300) and Ly6G (1:200). After rinses with PBS, the slides were incubated with Cy3-conjugated goat anti-rabbit IgG (H + L) at room temperature in the dark for 50 min. Nuclei were counterstained with 4′,6-diamidino-2-phenylindole (DAPI). Positive results were quantified using ImageJ software(Image J 2 system software https://imagej.net/Downloads).

### TUNEL staining

The TUNEL assay was used to detect apoptotic nuclei in lung sections according to the manufacturer’s instructions. In this assay, nuclei containing fragmented DNA displayed red fluorescence, while normal nuclei emitted the blue fluorescence of DAPI. Positive results were quantified using ImageJ software(Image J 2 system software https://imagej.net/Downloads).

### Western blot analysis

Lungs were extracted in RIPA lysis buffer containing a protease/phosphatase inhibitor cocktail and then centrifuged. Protein concentrations in the supernatants were measured using a BCA protein assay kit. Equal amounts of protein (40 μg) from each sample were resolved on 8% SDS-PAGE gels, and proteins were transferred to a 0.45 μm PVDF membrane. The membrane was blocked with 5% skim milk at room temperature for 1 h and then incubated overnight at 4 °C with primary antibodies against GAPDH (1:5000), JAK2 (1:1000), p-JAK2 (1:1000), STAT3 (1:2000), or p-STAT3 (1:2000). The membrane was washed three times for 10 min each with Tris-buffered saline containing 0.05% Tween-20 (TBST) and then incubated with the appropriate HRP-conjugated secondary antibodies (1:5000) for 1 h at room temperature. The membrane was washed again, and bands were revealed by incubating the membrane with the ECL solution in the dark. The blots were analysed using a LI-COR Odyssey Imaging System (LI-COR, Lincoln, NE, USA). The band densities were calculated using ImageJ software(Image J 2 system softwarehttps://imagej.net/Downloads).

### Immunohistochemical staining

Paraffin sections (4-μm-thick) were dewaxed with xylene and rehydrated in ethanol. Endogenous peroxidase activity was blocked by incubating sections with 0.3% hydrogen peroxide, and non-specific binding was blocked with normal goat serum for 30 min. The sections were then stained overnight with primary rabbit anti-p-JAK2 or anti-p-STAT3 antibodies, rinsed with PBS, and incubated with the HRP-conjugated secondary antibody for 30 min at room temperature. Colour development was achieved by incubating sections with the HRP substrate 3,5-diaminobenzidine. Positive staining was observed microscopically as brownish-yellow or tan signals, which were quantified using ImageJ software(Image J 2 system software https://imagej.net/Downloads).

### Image analysis

The quantitative analysis of western blots, immunohistochemical and immunofluorescence staining were used by ImageJ software(Image J 2 system software https://imagej.net/Downloads. Details about how the imaging program Image J are shown in supplementary material.

### Statistical analysis

All analyses were performed using GraphPad Prism 5 software (GraphPad Prism 5.01 http://www.xdowns.com/soft/xdowns2009.asp?softid=49668&downid=60&id=52443). Data are presented as the means ± standard deviations (SD). Histological scores were compared using the Mann–Whitney U test; all other data were analysed using one-way analysis of variance. *P* < 0.05 was considered significant.

## Results

### Daphnetin ameliorates pancreatic injury and inflammation in mice with SAP

**Figure 1:**
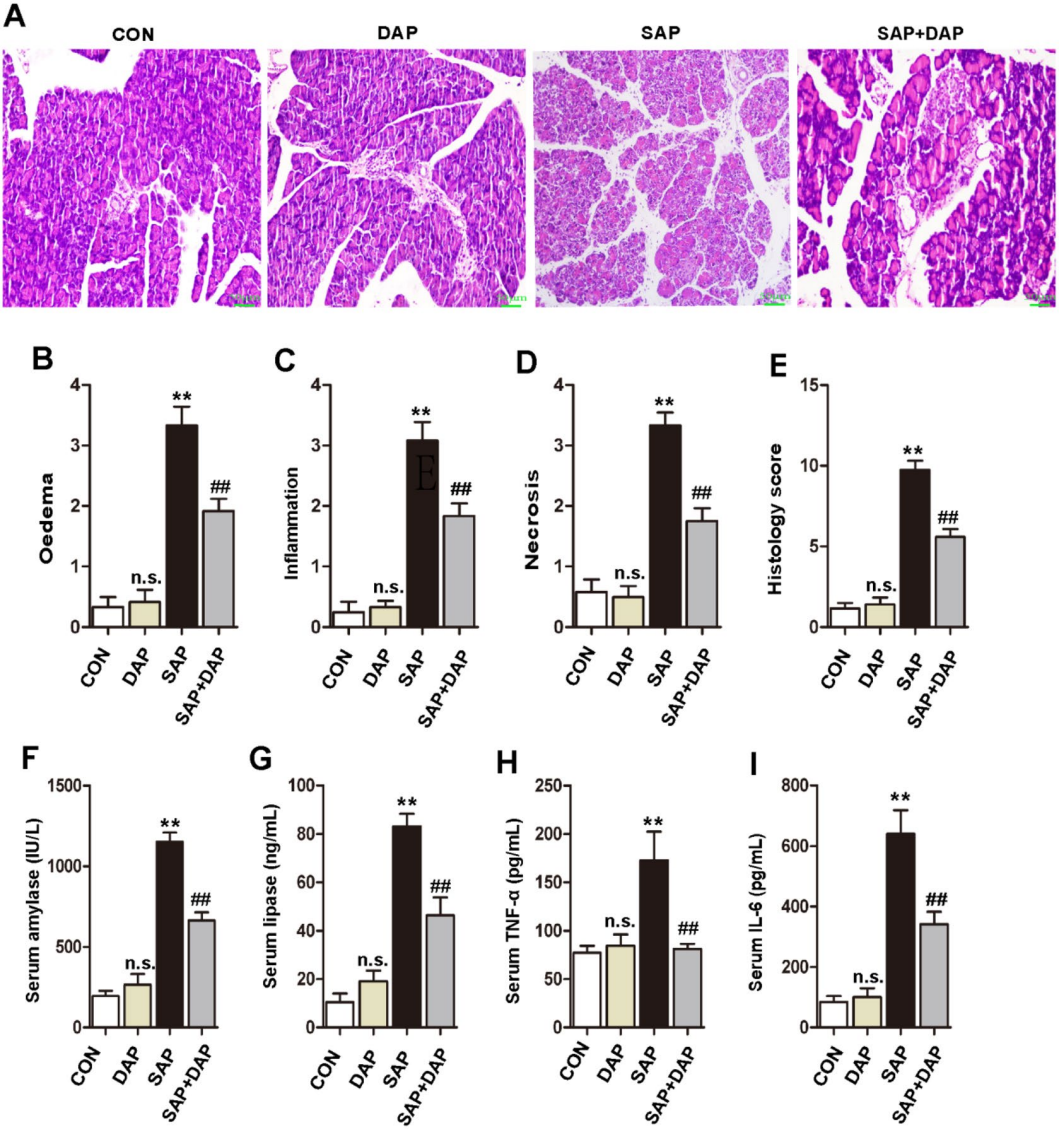
Daphnetin can ameliorate pancreatic injury and inflammation in mice with SAP. (**A**). Hematoxylin and eosin staining of the pancreas tissue, the magnification of each histological images were × 200. Scoring of (**B**) tissue edema, (**C**) inflammatory cell infiltration, (**D**) acinar cell necrosis, and (**E**) overall histopathological score were conducted blindly by experienced pathologists. Blood was collected 24 h after the first injection of l-arginine, the serum levels of amylase (**F**), lipase (**G**), TNF-α(H) and IL-6 (**I**) were detected by ELISA kits. The histograms were created using GraphPad 5 software(GraphPad Prism 5.01 http://www.xdowns.com/soft/xdowns2009.asp?softid=49668&downid=60&id=52443).CON group, l-arginine vehicle + daphnetin vehicle; SAP group, l-arginine + daphnetin vehicle; DAP group, l-arginine vehicle + daphnetin; SAP + DAP group, l-arginine + daphnetin. Data are presented as the mean ± SD. n = 6 /group. **P < 0.01 versus CON group; ^##^*P* < 0.01 versus SAP group, ^n.s.^*P* > 0.05 versus CON group. Scale bars: 50 μm.

Four groups of mice (n = 6) were administered the vehicle (CON), l-arginine alone (SAP), l-arginine plus daphnetin (SAP + DAP), or daphnetin alone (DAP). The severity of SAP was assessed by histological scoring of tissue oedema, inflammation, and necrosis. Compared with the CON and DAP mouse groups, the SAP mice displayed pancreatic tissue damage consisting of interstitial oedema, massive neutrophil infiltration, and necrosis (Fig. 1A). Notably, pancreatic injury in mice was significantly reduced by the daphnetin pretreatment (Fig. 1B–E). Serum amylase and lipase concentrations are considered the most sensitive and specific markers of pancreatitis^24^. Compared with the CON and DAP groups, the SAP group displayed significantly higher serum amylase and lipase levels, but these changes were markedly reduced by the daphnetin treatment (Fig. 1F,G). Serum amylase and lipase levels in the CON and DAP groups were not significantly different. Increases in serum and tissue concentrations of the cytokines TNF-α and IL-6 indicate localized and/or systemic inflammatory responses. Consistent with this finding, TNF-α (Fig. 1H) and IL-6 (Fig. 1I) levels were markedly increased in the SAP group compared with the CON and DAP groups, but the levels were significantly lower in the SAP + DAP group. Based on these results, daphnetin protected against pancreatic injury in the C57BL/6 mouse model of l-arginine-induced SAP.

### Daphnetin reduces lung damage and inflammation in mice with PALI

**Figure 2:**
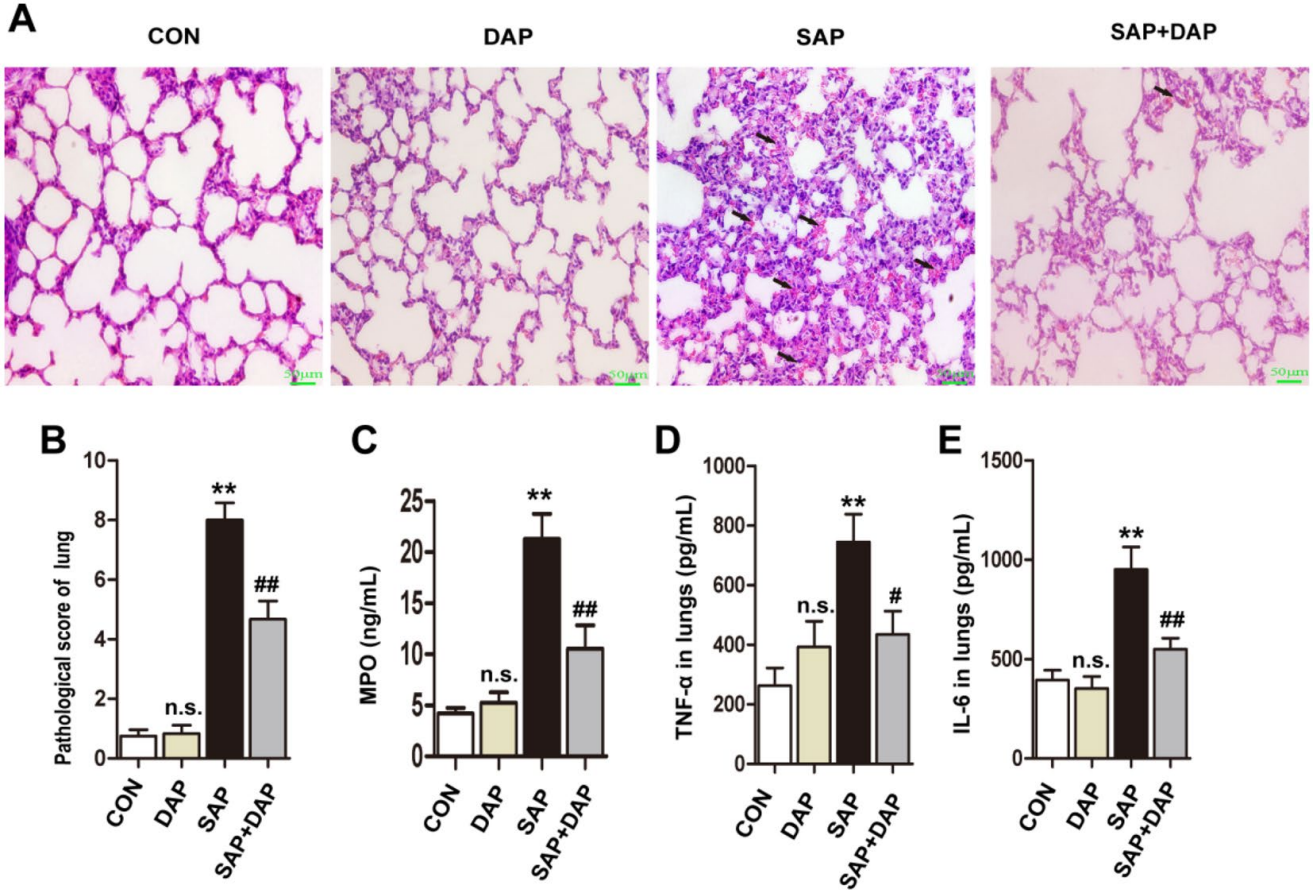
Daphnetin reduces lung damage and inflammation in mice with PALI. (**A**) Hematoxylin and eosin staining of the pancreas tissue, the magnification of each histological images were × 200. Arrows show alveolar thickening and inflammatory cell infiltration in lungs. (**B**) Scoring of alveolar septal capillary density in lung issues. Indicators of inflammation in lung tissue were detected by ELISA kits. The level of MPO (**C**) and TNF-α (**D**) and IL-6 (**E**) were reduced significantly after the pretreatment of daphnetin.The histograms were created using GraphPad 5 software(GraphPad Prism 5.01 http://www.xdowns.com/soft/xdowns2009.asp?softid=49668&downid=60&id=52443). CON group, l-arginine vehicle + daphnetin vehicle; SAP group, l-arginine + daphnetin vehicle; DAP group, l-arginine vehicle + daphnetin; SAP + DAP group, l-arginine + daphnetin. Data are presented as the mean ± SD. n = 6 /group. **P < 0.01 versus CON group. ^#^*P* < 0.05 versus SAP group; ^##^*P* < 0.01 versus SAP group, ^n.s.^*P* > 0.05 versus CON group. Scale bars: 50 μm.

Next, we determined whether daphnetin treatment attenuated PALI. HE staining of the lung tissue from mice in the SAP group revealed severe damage to the alveolar structure, massive neutrophil infiltration, and congestion of the alveolar septal capillaries, but the SAP-induced changes were milder in the SAP + DAP group (Fig. 2A). Accordingly, the lung histopathological score for the SAP + DAP group was significantly lower than that for the SAP group (Fig. 2B).

**Figure 3:**
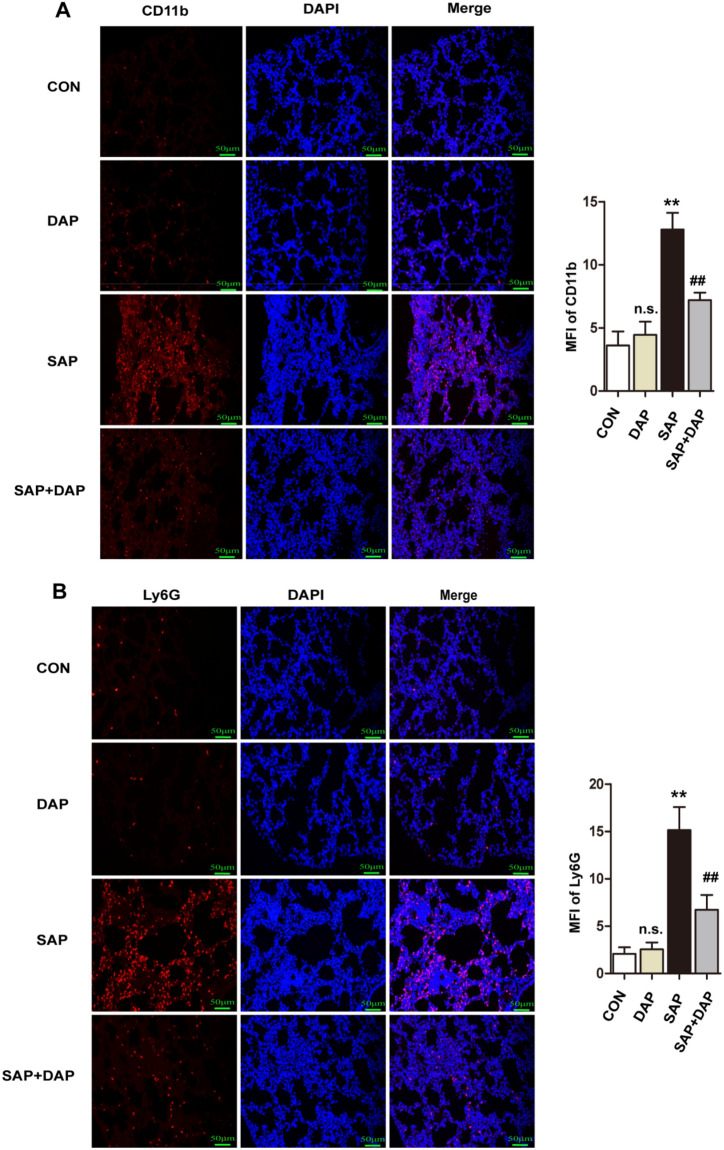
Daphnetin pretreatment inhibited the infiltration of macrophages and neutrophils. Representative images of immunofluorescence detection of the macrophages markers CD11b (**A**) and the neutrophil marker Ly6G (**B**) in the lung tissues. Nuclei were counterstained with DAPI. The results for the quantitation of mean fluorescence intensity (MFI) in the red channel are presented. The histograms were created using GraphPad 5 software(GraphPad Prism 5.01 http://www.xdowns.com/soft/xdowns2009.asp?softid=49668&downid=60&id=52443). The quantitative analysis of immunostaining was used by ImageJ software(Image J 2 system software https://imagej.net/Downloads). CON group, l-arginine vehicle + daphnetin vehicle; SAP group, l-arginine + daphnetin vehicle; DAP group, l-arginine vehicle + daphnetin; SAP + DAP group, l-arginine + daphnetin. Data are presented as the mean ± SD. n = 6 /group. **P < 0.01 vs. CON group. ##*P* < 0.01 versus SAP group, n.s. *P* > 0.05 versus CON group. Scale bars: 50 μm.

MPO activity is a marker of neutrophil infiltration, and elevated tissue levels of MPO, TNF-α, and IL-6 are thus indicative of tissue inflammation. Quantification of TNF-α, IL-6, and MPO levels in lung tissue extracts using ELISA revealed that all three inflammatory mediators were present at markedly higher levels in the lungs of mice in the SAP group than in the CON and DAP groups (Fig. 2C–E). Consistent with the histological analyses, significantly lower levels of all three inflammatory markers were detected in the lungs of mice pretreated with daphnetin before SAP induction (Fig. 2C–E). We performed immunofluorescence staining of lung tissue sections to detect macrophages (CD11b) and neutrophils (Ly6G) and to confirm these findings. As expected, the induction of PALI resulted in an increase in the numbers of macrophages and neutrophils in the lung sections, but the increased cell number was attenuated by the daphnetin pretreatment (Fig. 3). These data indicated that daphnetin ameliorated PALI.

### Daphnetin inhibits apoptosis in the lungs of mice with PALI

**Figure 4:**
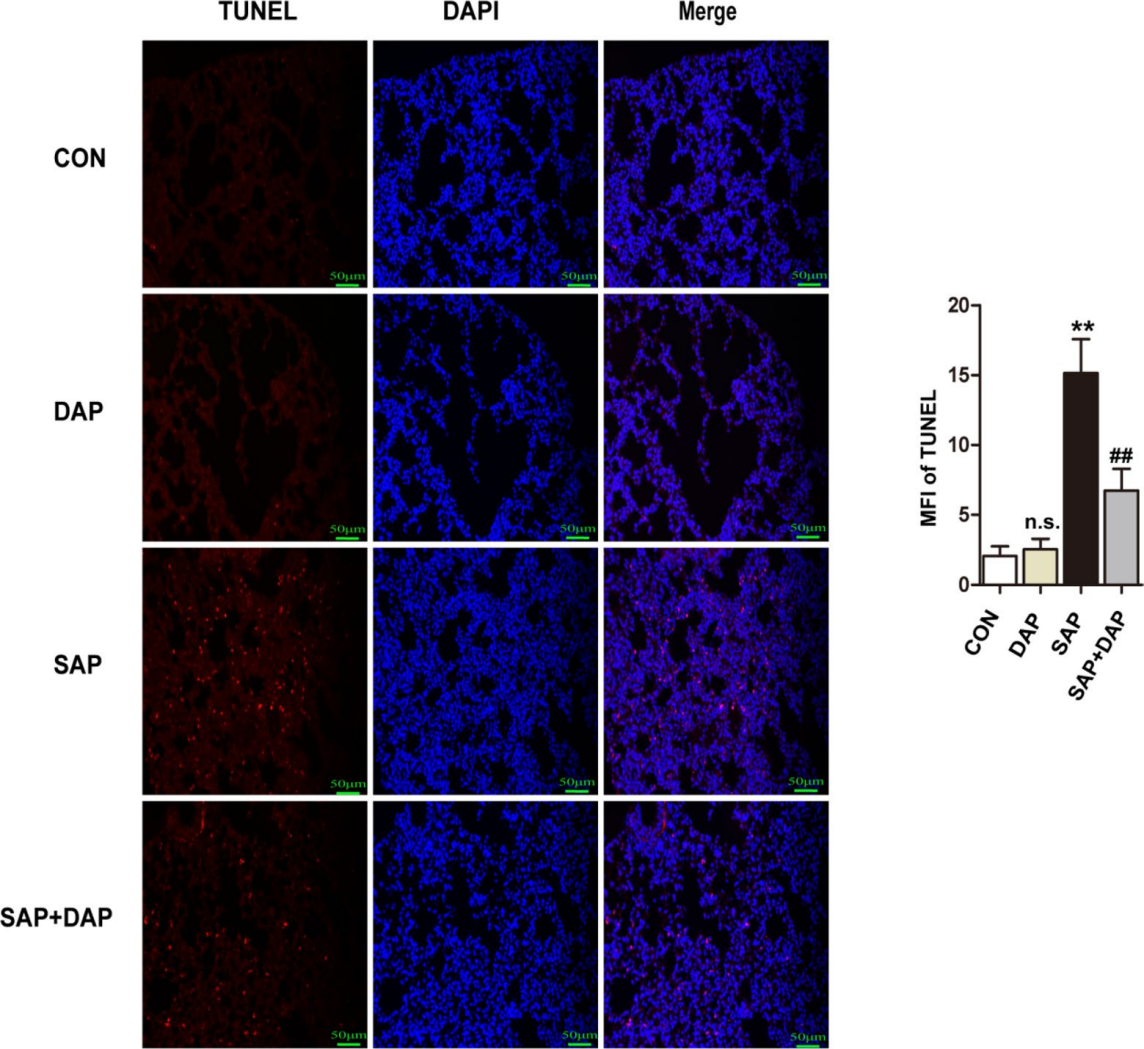
Daphnetin Pretreatment Inhibited the Apoptosis in Lung Tissues. Representative images of immunofluorescence detection of the apoptosis markers of TUNEL in the lung tissues. Nuclei were counterstained with DAPI. The results for the quantitation of mean fluorescence intensity (MFI) in the red channel are presented. The histograms were created using GraphPad 5 software(GraphPad Prism 5.01 http://www.xdowns.com/soft/xdowns2009.asp?softid=49668&downid=60&id=52443). The quantitative analysis of immunostaining was used by ImageJ software(Image J 2 system software https://imagej.net/Downloads). CON group, l-arginine vehicle + daphnetin vehicle; SAP group, l-arginine + daphnetin vehicle; DAP group, l-arginine vehicle + daphnetin; SAP + DAP group, l-arginine + daphnetin. Data are presented as the mean ± SD. n = 6/group. **P < 0.01 versus CON group. ^##^*P* < 0.01 versus SAP group, ^n.s.^*P* > 0.05 vs. CON group. Scale bars: 50 μm.

We performed TUNEL staining to determine whether daphnetin protected against PALI-induced apoptosis of lung cells. Indeed, apoptotic cells were more abundant in the lung tissues of mice in the SAP group than in the control groups, but the degree of apoptosis was significantly reduced by the daphnetin pretreatment (Fig. 4, Supplementary Figure S1A). Thus, daphnetin reduced PALI through both anti-inflammatory and anti-apoptotic effects on the lung.

### Daphnetin inhibits activation of the JAK2–STAT3 pathway in the lungs of mice with PALI

**Figure 5:**
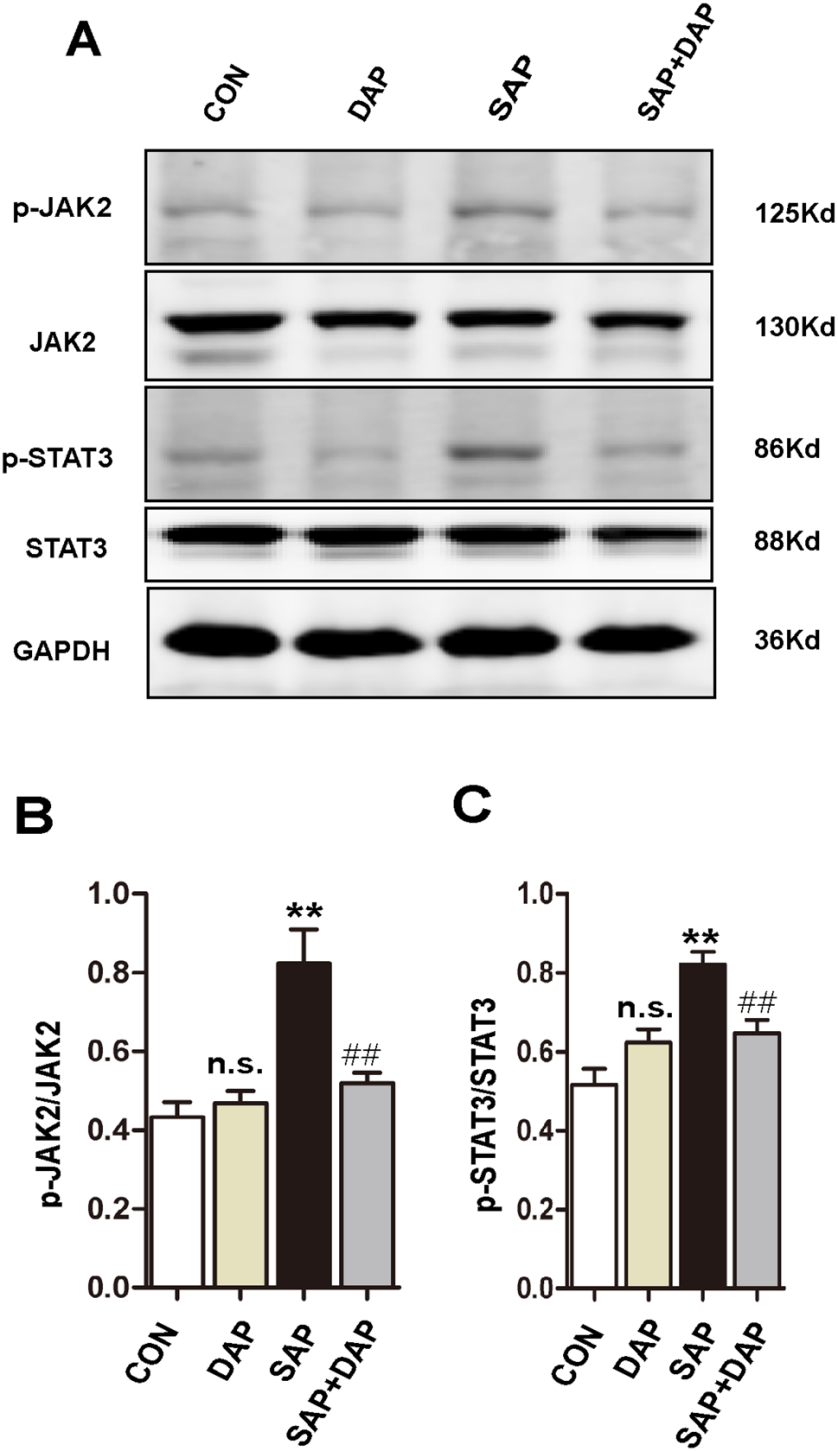
Daphnetin inhibits activation of the JAK2–STAT3 pathway in the lungs of mice with PALI. (**A**) Western blot analysis of p-JAK2, JAK, p-STAT3 and STAT3 expression in lung of each group, Bar graph showed the ratio of p-JAK2: total JAK2 protein (**B**). p-STAT3: total STAT3 protein (**C**). Full-length blots are presented in Supplementary Figure 2. The data are presented as the means ± SD. The histograms were created using GraphPad 5 software(GraphPad Prism 5.01 http://www.xdowns.com/soft/xdowns2009.asp?softid=49668&downid=60&id=52443). The quantitative analysis of blots was used by ImageJ software(Image J 2 system software https://imagej.net/Downloads). Full-length blots are presented in Supplementary Figure 2. CON group, l-arginine vehicle + daphnetin vehicle; SAP group, l-arginine + daphnetin vehicle; DAP group, l-arginine vehicle + daphnetin; SAP + DAP group, l-arginine + daphnetin. n = 3 /group, data are representative of three independent experiments. **P < 0.01 versus CON group; ^##^*P* < 0.01 versus SAP group, ^n.s.^*P* > 0.05 versus CON group.

The JAK–STAT signalling pathway has been widely reported to play an important role in lung injury^13–25^. We performed western blot analyses of total and activated (phosphorylated) JAK2 and STAT3 expression in lung extracts to better understand the mechanism by which daphnetin reduces SAP-associated tissue damage in the lung. As shown in Fig. 5A (Full-length blots are presented in Supplementary Figure 2), total JAK2 and STAT3 expression was comparable in lung tissue from mice in the CON, DAP, SAP, and SAP + DAP groups. However, the levels of p-JAK2 and p-STAT3 were increased in the SAP group compared with the control groups, but the increase was attenuated by the daphnetin pretreatment. Accordingly, quantification of the band densities revealed that the ratios of p-JAK2:total JAK2 protein and p-STAT3:total STAT3 protein were significantly lower in the SAP + DAP group than in the SAP group (Fig. 5B,C).

**Figure 6:**
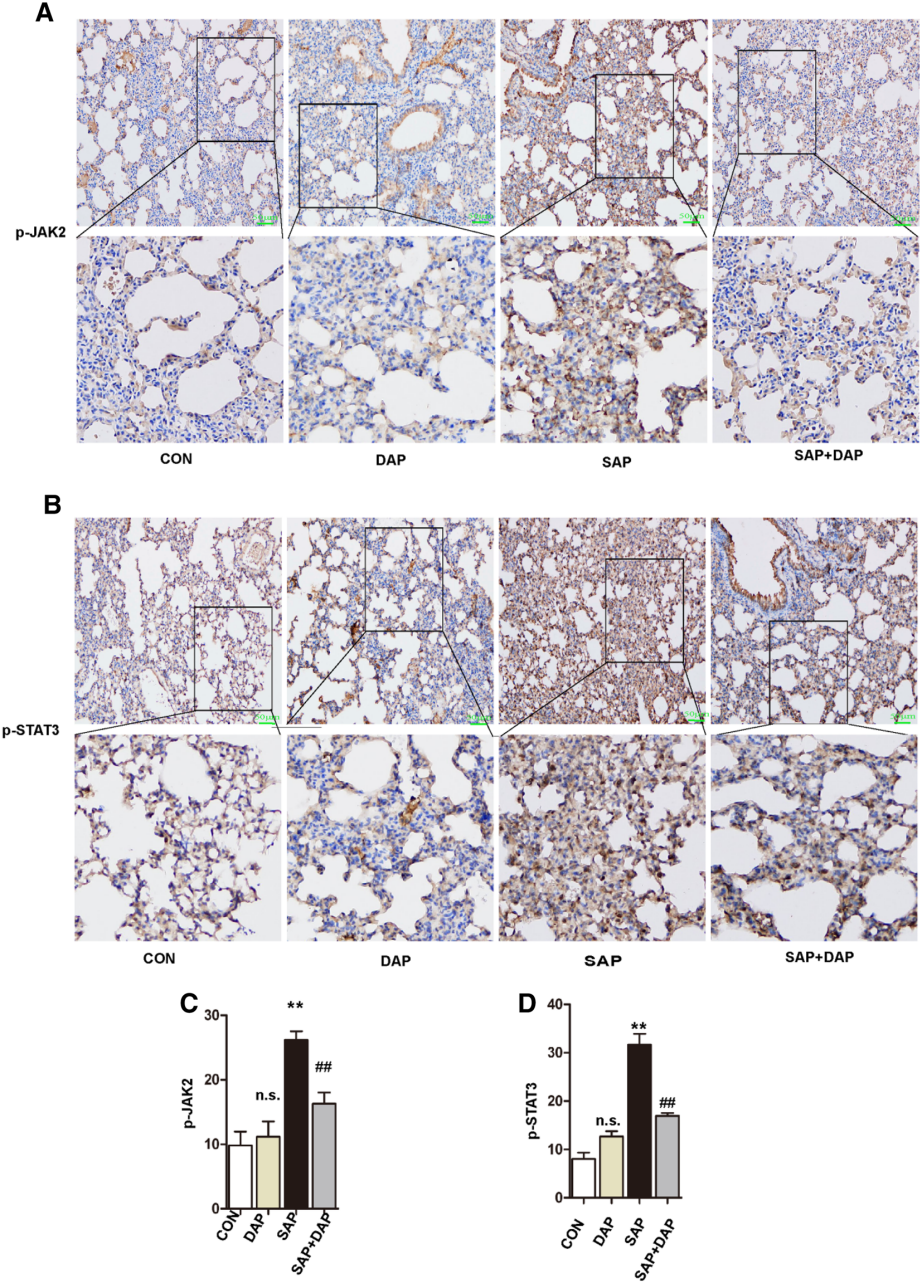
Daphnetin inhibits activation of the JAK2–STAT3 pathway in the lungs of mice with PALI. Immunohistochemical staining of p-JAK2 (**A**) and p-STAT3 (**B**) in lung of each group. The magnification of each histological images were × 200. Quantitative analysis of p-JAK2 (**C**) and p-STAT3 (**D**). The data are presented as the means ± SD. The histograms were created using GraphPad 5 software (GraphPad Prism 5.01 http://www.xdowns.com/soft/xdowns2009.asp?softid=49668&downid=60&id=52443). The quantitative analysis of immunostaining was used by ImageJ software(Image J 2 system software https://imagej.net/Downloads). CON group, l-arginine vehicle + daphnetin vehicle; SAP group, l-arginine + daphnetin vehicle; DAP group, l-arginine vehicle + daphnetin; SAP + DAP group, l-arginine + daphnetin. **P < 0.01 versus CON group; ^##^*P* < 0.01 versus SAP group, n.s. *P* > 0.05 versus CON group. Scale bars: 50 μm.

We performed immunohistochemical staining for p-JAK2 and p-STAT3 in lung sections from the four mouse groups to confirm these results. Consistent with the western blot analyses, the expression of p-JAK2 (Fig. 6A,C, Supplementary Figure S1B) and p-STAT3 (Fig. 6B,D) was significantly lower in tissues from the SAP + DAP group than in tissues from the SAP group.

Considering the anti-inflammatory effect of daphnetin on the reduction of systemic inflammation in lung tissue and subsequent effect on JAK2–STAT3 pathway, we chose human lung cells (A549) to conducted an in vitro experiment. The activation of JAK2-STAT3 signalling in LPS-induced lung cells was inhibited by the daphnetin pretreatment, showing that the protective effect of daphnetin on the lung tissue may be mediated by the JAK2-STAT3 signalling pathway (Supplementary Fig. 3,4). Collectively, these results suggest that daphnetin reduces lung damage in mice with PALI probably by inhibiting the activation of the JAK2–STAT3 signalling pathway, thereby reducing the extent of inflammation and cell apoptosis in the lung.

## Discussion

The treatment of PALI and other disorders occurring secondary to SAP has been clinically challenging, mainly because the underlying pathophysiological mechanisms are still poorly understood^26^. Chinese herbs have been widely used in Asia for hundreds of years. However, scientific data to support the clinical efficacy of these traditional Chinese medicines are lacking. Moreover, while previous studies have shown that daphnetin exerts a range of anti-inflammatory effects on acute liver failure^26^, experimental colitis^27^, and encephalomyelitis^28^, little is known about the precise molecular mechanisms that mediate the beneficial effects of daphnetin.

In this study, a PALI mouse model was used to study the effects of daphnetin on the pancreas and lung tissue. Daphnetin reduced not only SAP-associated pancreatic and lung tissue injury but also the accompanying inflammatory response, as evidenced by the levels of tissue and serum markers. Tissue accumulation of macrophages and neutrophils, serum and lung levels of TNF-α and IL-6, and the degree of cell apoptosis were reduced in mice pretreated with daphnetin before SAP induction. In addition, the daphnetin pretreatment inhibited activation of the JAK2–STAT3 pathway in models of lung injury in vivo and in vitro. To our knowledge, this report is the first to show that daphnetin is an effective treatment for pancreatitis-associated lung injury.

Although the pathogenesis of PALI is unknown, dysregulation of the inflammatory response is generally believed to lead to organ damage and death^29^ by inducing oxidative stress and apoptosis^30^. Exposure of cells to external stressors stimulates a response involving the release of many inflammatory factors, including TNF-α and IL-6. These cytokines are essential regulators of neutrophil function^31^, and increased levels are associated with the severity of PALI^32^. Our findings that daphnetin significantly reduced pancreas and lung tissue injury, inhibited IL-6 and TNF-α production, and decreased MPO activity in mice with SAP are consistent with a previous study by Shen et al. (2017), who showed that daphnetin exerts anti-inflammatory and protective effects on a mouse model of endotoxin-induced lung injury^33^.

The JAK2–STAT3 pathway plays an indispensable role in cell growth, differentiation, proliferation, and apoptosis^34^ and mediates the response of cells to cytokines^35^. Piao et al. (2019) found that inactivation of JAK2 and STAT3 phosphorylation significantly inhibited inflammation and oxidative stress, thereby ameliorating lung tissue damage in a mouse model^14^. Here, we observed significantly increased phosphorylation of JAK2 and STAT3 in the lungs of mice with SAP, and this change correlated with the effects on serum TNF-α, IL-6, amylase, and lipase levels. Notably, each of these effects was reduced in mice pretreated with daphnetin, consistent with a report by WW et al. (2014) showing that daphnetin protects against lipopolysaccharide-induced lung inflammation and injury in mice^36^. A similar inhibitory effect of the daphnetin pretreatment on the JAK2-STAT3 pathway was observed in the lung cell injury model. We speculate that JAK2–STAT3 signalling might be a critical intermediary pathway that mediates the anti-inflammatory effect of daphnetin on PALI. Moreover, apoptosis of pulmonary microvascular endothelial cells is presumed to be associated with increases in lung permeability^36^, and we found that daphnetin reduced cell apoptosis in the lung. However, further mechanistic studies will be necessary to confirm this specific link.

Our study has some limitations. First, we employed an l-arginine-induced model of SAP, and the results should be verified in other SAP models in future experiments. Second, we pretreated the mice with daphnetin before the induction of SAP, which is inconsistent with the clinical situation. The initiation of daphnetin treatment at other time points relative to SAP induction will be examined in future experiments. Finally, many other signalling pathways are involved in PALI, such as the Nrf2 signalling pathway^38^ and NF-κB signalling pathway. In this study, we justified that daphnetin inhibited the activation of JAK2–STAT3 signalling. We did not clearly determine whether daphnetin influenced other signalling pathways.

In summary, daphnetin preconditioning prior to l-arginine-induced SAP reduces acute lung injury by reducing the infiltration of inflammatory cells and inhibiting inflammatory cytokine secretion and cell apoptosis in the lung tissue. Daphnetin may exert these effects by inhibiting the activation of JAK2-STAT3 signalling, which provides a new perspective for the study of its molecular mechanism.

### Statement

The study was carried out in compliance with the ARRIVE guidelines.

## Supplementary Information

Supplementary Information.
