# Supplementary material for: Daphnetin ameliorates acute lung injury in mice with severe acute pancreatitis by inhibiting the JAK2–STAT3 pathway
Source: Sci Rep. 2021 Jun 1;11:11491. doi: 10.1038/s41598-021-91008-6 (PMC8169853; doi:10.1038/s41598-021-91008-6)
Supplement: Supplementary file 1 — Supplementary Information. [file 41598_2021_91008_MOESM1_ESM.pdf]

Daphnetin Ameliorates Acute Lung Injury in Mice with Severe Acute Pancreatitis by Inhibiting the JAK2–STAT3 Pathway

Shujun Yang[1,2,3#], Yaodong Song[1,2,3#], Qiaofang Wang[1,2], Yanna Liu[1,2], Zhongwei Wu[1], Xiaojia Duan[1], Yan Zhang[1], Xiuqian Bi[1], Yuanzhang Geng[1], Sanyang Chen[1], Changju Zhu[1,2,3*]

1 Department of Emergency, The First Affiliated Hospital of Zhengzhou University, No 1 Eastern Jianshe Road, Zhengzhou, 450052, Henan, China

2 Henan Key Laboratory of Emergency and Trauma Research Medicine, Henan, China

3 Key Laboratory of Hepatobiliary and Pancreatic Surgery and Digestive Organ Transplantation of Henan Province, China

* Corresponding Author： Changju Zhu

Email: zhuchangju98@163.com

# Joint first author: SY and YS contribute equally to this study.

Supplementary Methods

Daphnetin inhibits the activation of JAK2-STAT3 signaling pathway in human lung cells(A549) induced by LPS.

Human lung carcinoma type II epithelium-like A549 cells were purchased from the American Type Culture Collection(VA, USA). LPS was purchased from Beyotime Biotechnology(S1732). A549 cells (1×10^4 cells per well) were seeded overnight in 96-well tissue culture plates. Then A549 cells were treated with daphnetin(2.5, 5,10 or 20 μM) for 24h, and Cell viability was determined by the Cell Counting Kit-8(CCK8) assay. Cell viability showed that exposure of A549 cells to daphnetin at different concentrations for 24 h did not affect cell viability(Supplementary Fig. 1A). We choose 20 μM for our subsequent experiments according to the method of Chuang, C. et al[1]. A549 cells were seeded at a density of 3×10^5 cells/well in 6-well plates and cultured overnight. There are four groups in cell assay: (n=3): (i) a CON group (LPS vehicle + daphnetin vehicle), (ii) DAP group (LPS vehicle + daphnetin), (iii) LPS group (LPS + daphnetin vehicle) and (iv) LPS + DAP group (LPS + daphnetin). A549 cells were treated with Daphnetin (20 μM) for 0.5 h, followed by 24 h treatment with LPS (1 μg/mL)[2]. Cells were collected and JAK2, p-JAK2, STAT3, and p-STAT3 levels were examined by Western blotting.

Image Analysis

The quantitative analysis of western blots, immunohistochemical and immunofluorescence staining were used by ImageJ software(Image J 2 system software https://imagej.net/Downloads). The tools and steps we used were as follows: For western blots analysis: Open the ImageJ software and find images. Open “Image” in the menu bar and set Type to 8-bit. Click “Subtract Background” in Process to eliminate the effect of the image background. Click “Invert” under the Edit bar to

convert the WB blots to a bright band. Select the circular tool to circle all strips as much as possible, and click the "Measurement" button that appears under the Analyze menu bar to measure the gray statistics.

For immunohistochemical and immunofluorescence staining analysis: Import the Image you want to count in Image J. Click "Image" and change the RGB Color to the RGB Stack. Adjust the appropriate contrast. Click "Adjust" in the "Image" menu bar and select "Threshold". Change the B&W in the "Default" toolbar to Red. Select the positive signal area. Then click "Area Fraction" under the Set Measurement menu bar to set the calculation parameters, and click "Measure" under the Analyze menu bar to statistic the positive results.

References:

1. Shen, L. et al. Daphnetin Reduces Endotoxin Lethality in Mice and Decreases LPS-induced Inflammation in Raw264.7 Cells Via Suppressing JAK/STATs Activation and ROS Production. *Inflamm. Res.* **66**, 579-589 (2017).
2. Chuang, C. et al. Lipopolysaccharide Induces Apoptotic Insults to Human Alveolar Epithelial A549 Cells through Reactive Oxygen Species-Mediated Activation of an Intrinsic Mitochondrion-Dependent Pathway. *Arch. Toxicol.* **85**, 209-218 (2011).

Supplementary Figure Legends

Supplementary Figure S1.

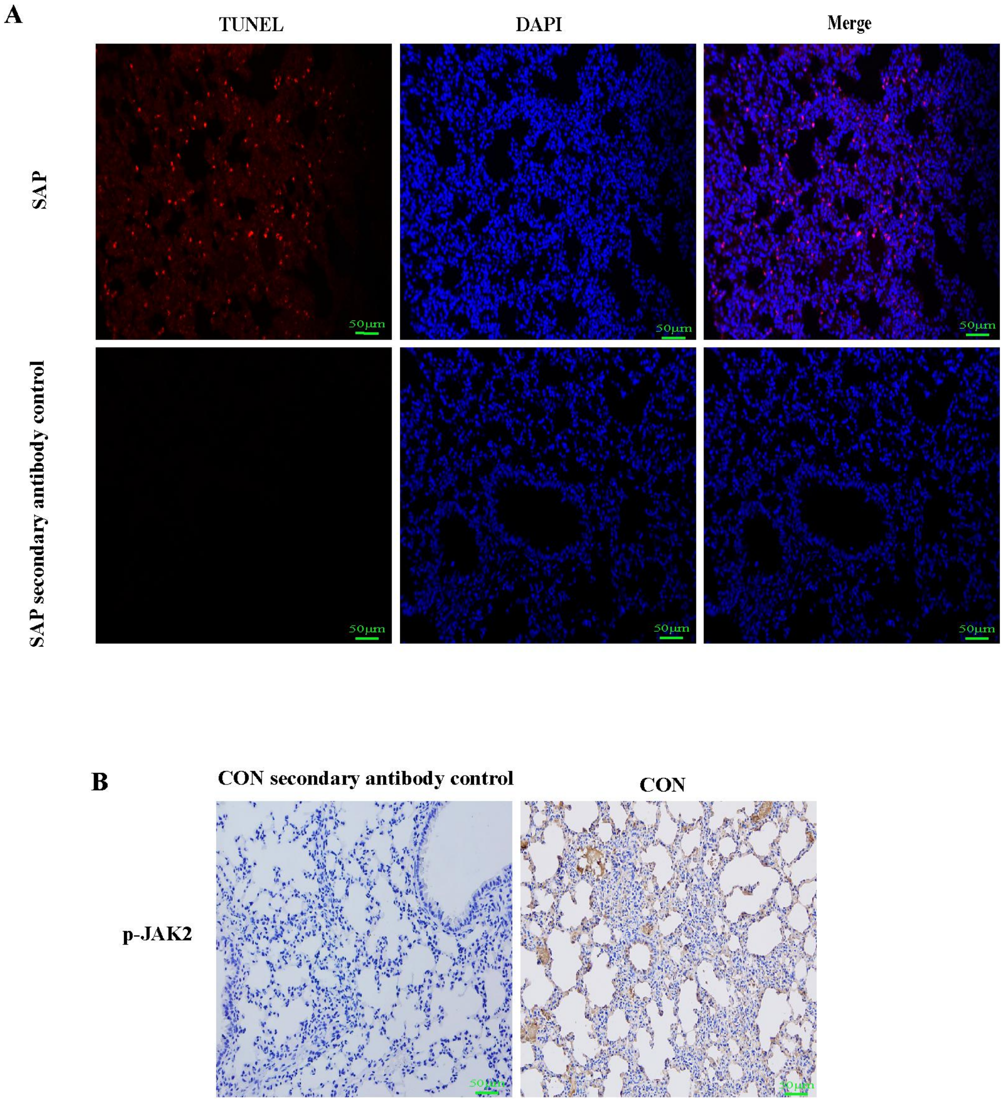

Supplementary Figure S1. The secondary antibody control of immunofluorescence staining(A) and immunohistochemical staining(B). The magnification of each histological images were×200. Scale bars: 50 μm.

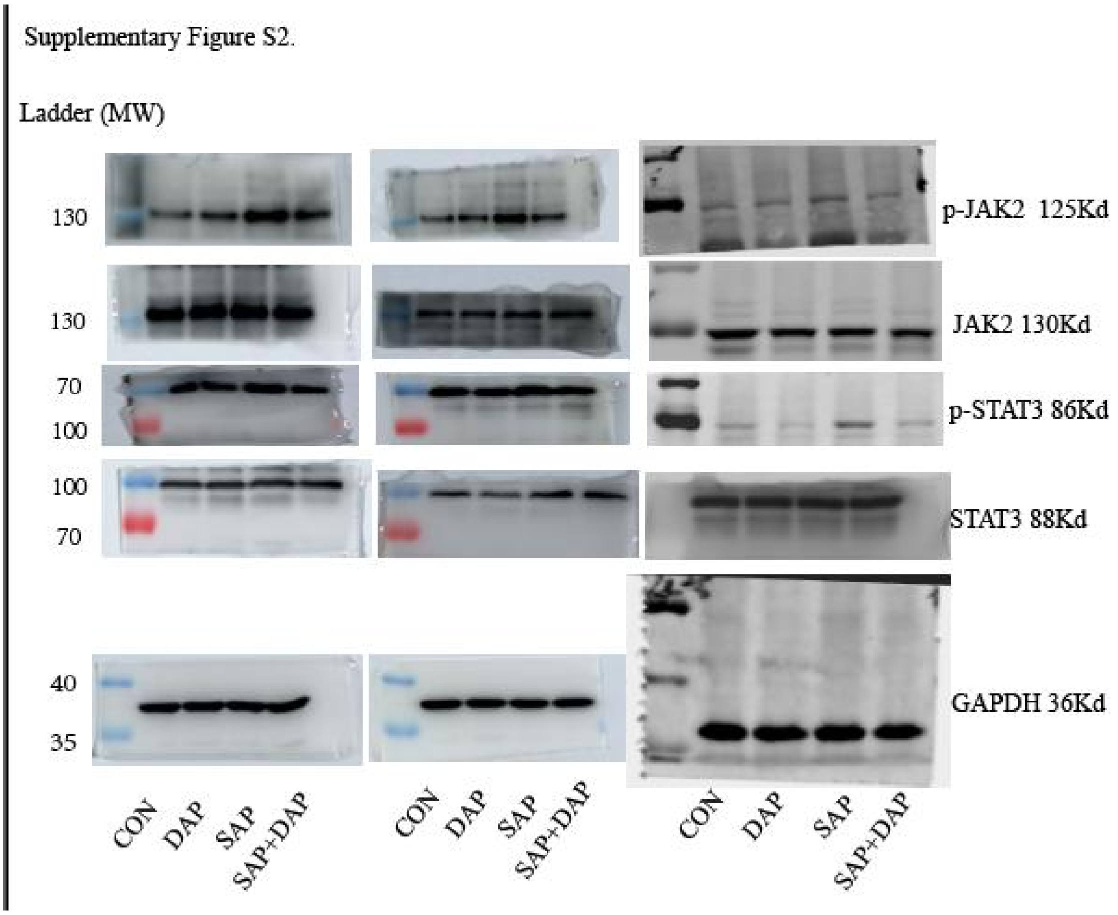

Supplementary Figure S2. Daphnetin inhibits activation of the JAK2–STAT3 pathway in the lungs of mice with PALI. Representative WB analysis of JAK2-STAT3 in lung issues. n=3. Data are representative of three independent experiments.

Supplementary Figure S3.

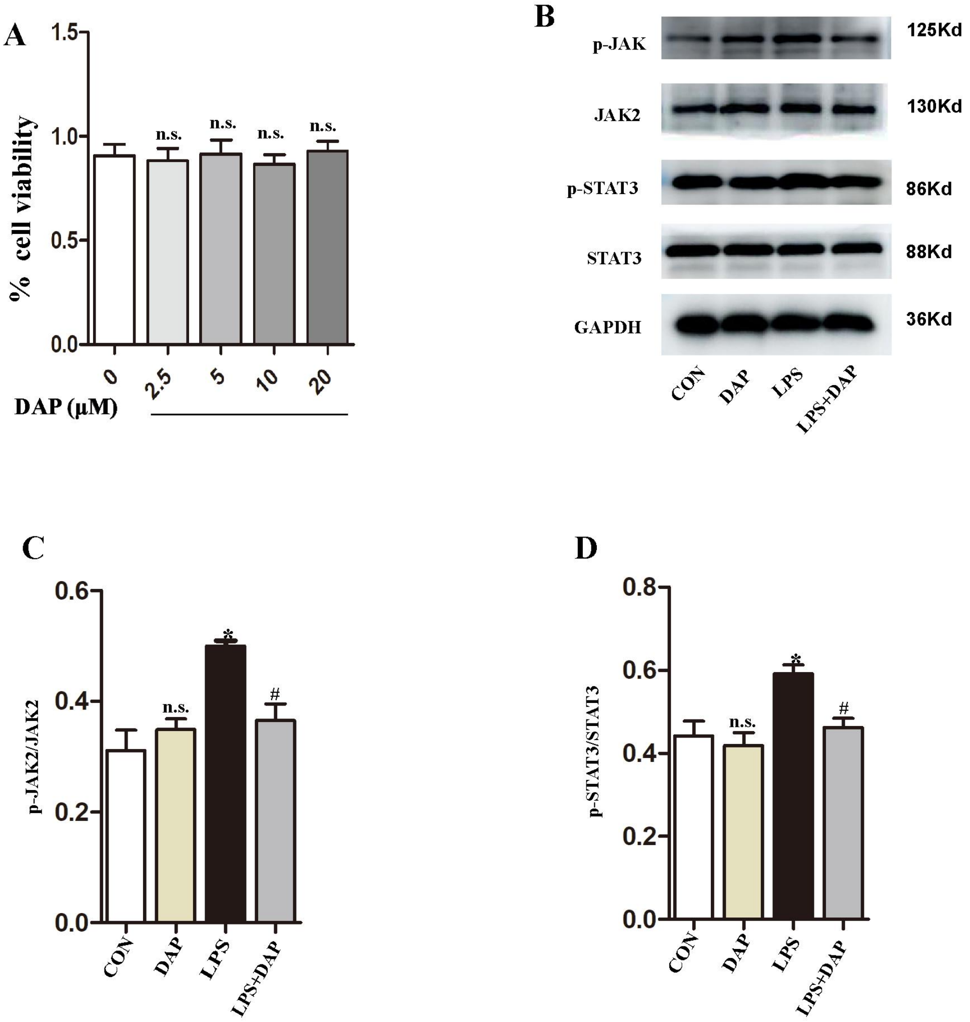

Supplementary Figure S3. Daphnetin inhibits the activation of JAK2-STAT3 signaling pathway in human lung cells(A549) induced by LPS. (A) Viability of A549 cells, that were treated with daphnetin at the dose indicated for 24h, was analyzed by the CCK8 assay. (B) Western blot analysis of p-JAK2, JAK, p-STAT3 and STAT3 expression in A549 cells. (C) The ratio of p-JAK2: total JAK2 protein. (D)The ratio of p-STAT3: total STAT3 protein. The data are presented as the means ± SD. Full-length blots are presented in Supplementary Figure 4. The data are presented as the means ± SD. The histograms were created using GraphPad 5 software(GraphPad Prism 5.01 http://www.xdowns.com/soft/xdowns2009.asp?softid=49668&downid=60&id=52443). The quantitative analysis of blots was used by ImageJ software(Image J 2 system software https://imagej.net/Downloads). Full-length blots are presented in Supplementary Figure 4. *P<0.01 vs. CON group; $^{\#}$P<0.01 vs. LPS group, n.s.P>0.05 vs. CON group. n=3. Data are representative of three independent experiments.

Supplementary Figure S4.

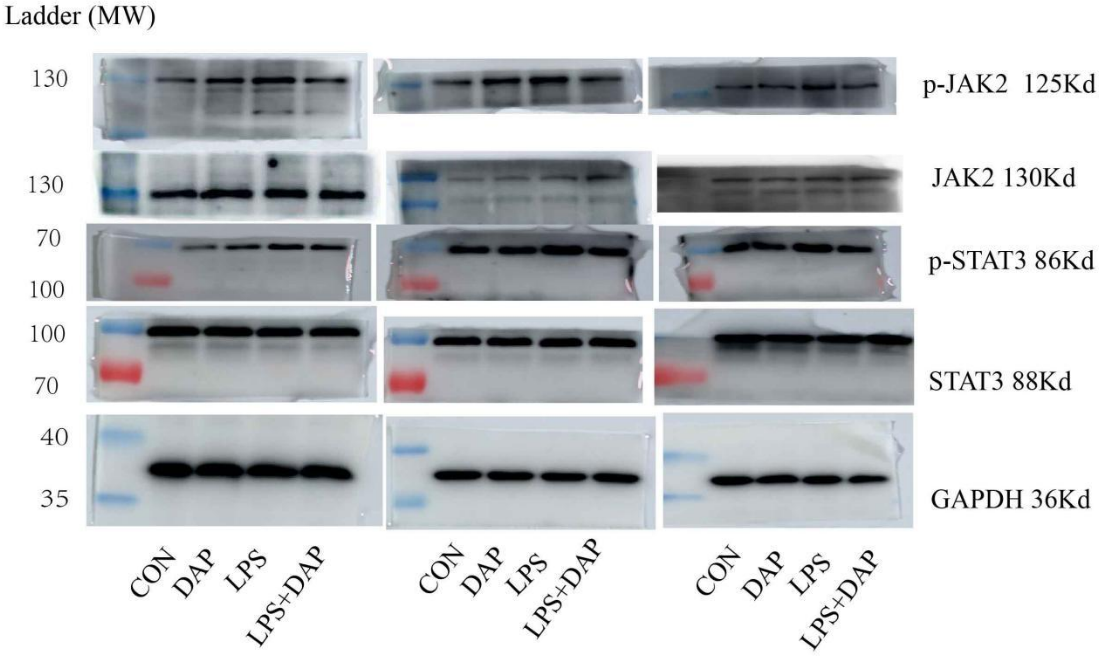

Supplementary Figure S4. Daphnetin inhibits activation of the JAK2–STAT3 pathway in A549 cells. Representative WB analysis of JAK2-STAT3 in A549 cells. n=3. Data are representative of three independent experiments.
